# Massive Uterine Leiomyoma in a Phenotypic Male

**DOI:** 10.7759/cureus.62977

**Published:** 2024-06-23

**Authors:** Rohma Qureshi, Ahsan Shafiq, Jawayria Sajid, Amara Younas, Roshan Butt

**Affiliations:** 1 General Surgery, Shalamar Hospital, Shalamar Medical & Dental College, Lahore, PAK; 2 Surgical Oncology, Shaukat Khanum Memorial Cancer Hospital and Research Center, Lahore, PAK; 3 Surgical Oncology, Shalamar Hospital, Shalamar Medical & Dental College, Lahore, PAK; 4 Oncology, Shalamar Hospital, Shalamar Medical & Dental College, Lahore, PAK; 5 Surgery, Services Hospital Lahore, Lahore, PAK

**Keywords:** large abdominal mass, sexual differentiation, undescended testes, phenotypical male, virilization, ambiguious genitalia, massive leiomyoma, female prostate, cah, pseudohermaphrodistism

## Abstract

We present a case report of a 55-year-old male patient with congenital adrenal hyperplasia (CAH) and a large neoplastic mass in the abdomen. The patient presented with an abdominal mass and discomfort, along with a bilateral empty scrotum since birth. A diagnostic workup revealed the mass to be a uterine leiomyoma associated with CAH, a simple virilizing type. Treatment involved an exploratory laparotomy and excision of the mass, including the removal of the entire uterus. Complete removal of the mass and uterus was ensured. The patient's response to treatment was satisfactory. This case highlights how pre-operative and post-operative diagnoses can vary, along with the importance of early diagnosis of CAH and disorders of sexual differentiation (DSD), emphasizing the significance of unusual presentations and resultant complications, as they might go unnoticed. CAH in XX females may have unusual presentations, such as short stature and a male phenotype (Prader 5). The patient exhibited a normal pattern of male sexual function. This condition might go unnoticed, resulting in leiomyoma, adrenal tumors, prostate tumors if prostate tissue is present, and so on. Healthcare providers must watch out for such rare presentations.

## Introduction

Congenital adrenal hyperplasia (CAH) encompasses genetically determined steroidogenesis disorders, leading to cortisol and/or aldosterone deficiency and androgen excess, causing metabolic and virilizing effects [1]. We report a rare case of a giant uterine leiomyoma, 270 x 200 x 120 mm with associated CAH, highlighting the need for CAH screening and awareness of associated disorders. Elevated testosterone levels may increase leiomyoma risk [2-4]. CAH can cause masculinization, from hirsutism to virilized external genitalia, with genetic factors involving estrogen and progesterone receptors highly expressed [3]. Untreated CAH in XX individuals may induce leiomyoma due to elevated hormones [5]. Ideally, a multidisciplinary approach is required for these individuals, including early surgical intervention [6,7]. High androgen levels may stimulate estrogen-dependent organ growth, emphasizing CAH treatment (glucocorticoids) and screening to prevent complications [4,8]. Prenatal diagnosis by chorionic villus sampling (CVS) may be beneficial [1].

## Case presentation

Patient presentation

A 55-year-old male presented to the surgical outpatient clinic with complaints of abdominal distension, obstructive symptoms, abdominal tightness/heaviness particularly in the lower part, along with pain for the last two months. No aggravating or relieving factor was reported, and this was the first time the patient experienced this. The patient is reported to have had an empty scrotum since birth, which was also confirmed on examination. He has been married for 25 years, with no children. Sexual activity is normal. He has eight siblings, with him being the only one with this condition. the patient is reported to have some cousins with similar situations, but they have never been evaluated. CT abdomen with contrast was done, which revealed a large, circumscribed mass (thought to arise from the undescended testes) in the abdomen, along with bilateral adrenal hyperplasia [5,7,9,10]. Positive Barr bodies indicated the biological sex of the patient to be female [11,12]. This test was done after the surgical resection of the mass. Following is a summary of the patient's lab investigations (Table 1).

**Table 1:** Lab investigations Abbreviations: LDH: Lactate Dehydrogenase, AFP: Alpha Feto Protein, HB: Hemoglobin, TLC: Total Leukocyte Count, BSR: Blood Sugar Random, ALT: Alanine Aminotransferase, AST: Aspartate Aminotransferase, GGT: Gamma Glutamyl Transferase, ALP: Alkaline Phosphatase, C/E: Complete Examination The given table is the original work of the authors and has not been sourced from elsewhere.

Labs	Values	Normal value
Cortisol	10.88 mcg/dL AT 6PM	3-16 mcg/dL
LDH	631 U/L	135-275 U/L
B HCG	<2 mIU/mL	<10 mIU/mL
AFP	1.8 ng/mL	<8.5 ng/mL
Hb	19.1 g/dL	14-18 g/dL
TLC	11.02 x10^3/µL	4-11 x10^3/µL
Platelets	462/µL	150-450/µL
Hematocrit	56.7%	42-47%
BSR	115 mg/dL	<140 mg/dL
LFTS: ALT	13 U/L	5-40 U/L
AST	25 U/L	5-40 U/L
GGT	31 U/L	5-50 U/L
ALP	95 U/L	40-135 U/L
Albumin	4.5 g/dL	3.5-5.0 g/dL
Bilirubin	0.6 mg/dL	0.1-1.1 mg/dL
Total proteins	8.6 g/dL	6.3-8.3 g/dL
Urine C/E	8-12 pus cells/HPF	-
Barr bodies	+	-

Examination findings

The patient is 3 feet 10 inches tall and weighs 45 kg. He presents with a pulse of 74 bpm, blood pressure of 170/100 mmHg, and an SpO2 of 99%. Examination of the abdomen reveals it to be bulky, non-tender, and soft, with a mass effect felt in the hypogastrium. Despite normal sexual drive and arousal, as well as normal maintenance of erections and ejaculation patterns, he exhibits some atypical secondary sex characteristics. Puberty onset was at 17 years of age, his voice remains slightly high-pitched, and while axillary and pubic hair growth is normal, facial hair is also normal, and he does have male pattern baldness. There is no breast development. It is noted that androgen excess can cause accelerated skeletal maturation and reduced adult height [13,14].

Preoperative differential diagnoses

CAH with a tumor of the undescended testes, or testicular adrenal rest tumor (TART) syndrome. TART prevalence in male CAH patients is 40%, is the leading cause of infertility, and is challenging to distinguish from Leydig cell tumors [14]. After a multidisciplinary team meeting with radiology, oncology, surgical oncology, general surgery, hematology, and pathology, an exploratory laparotomy was planned. The procedure was done under general anesthesia (GA), and a large circumscribed abdominal mass was seen, along with what appeared to be a rudimentary organ resembling a uterus [9,10].

Postoperative diagnosis

The postoperative diagnosis was leiomyoma uteri with CAH. The biopsy results showed the absence of male gonads and identified rete of unspecified origin. The prostate is identified along with ovaries, uterus, and fallopian tubes, i.e., rudimentary Müllerian remnants [5]. The patient was labeled to be a pseudohermaphrodite with external and internal genitalia opposite to each other [7]. A genetic workup revealed Barr bodies in the buccal smear of the patient. Barr bodies are only positive in 39% of females and 1% of males [11], i.e., low sensitivity but high specificity. Barr bodies are defined as inactivated X chromosomes present in females with more than one X chromosome [11]. In XX disorders of sexual differentiation (DSD), affected females, the virilization can be so extensive that the girl can appear to be a true male with bilateral undescended testes [1,6,7], as was a case reported in Ghana [8].

**Figure 1:**
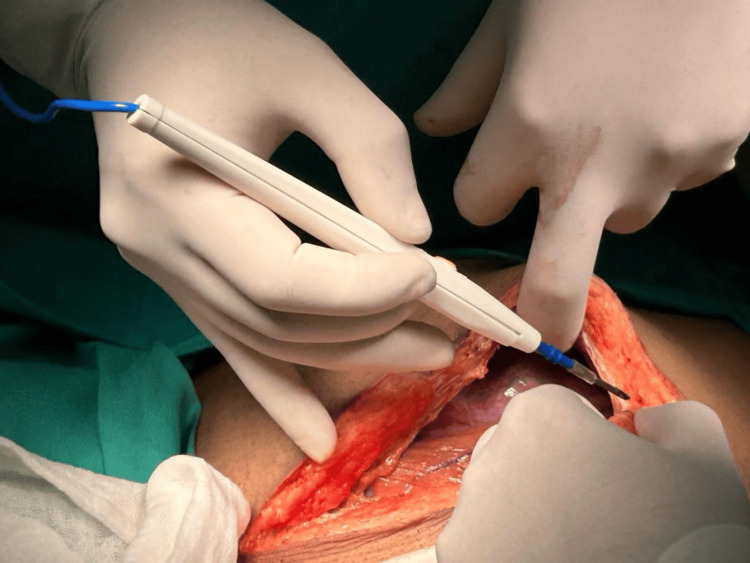
Explorative laparotomy showing a single large cut incision The given figure is the original work of authors and has not been sourced from elsewhere.

**Figure 2:**
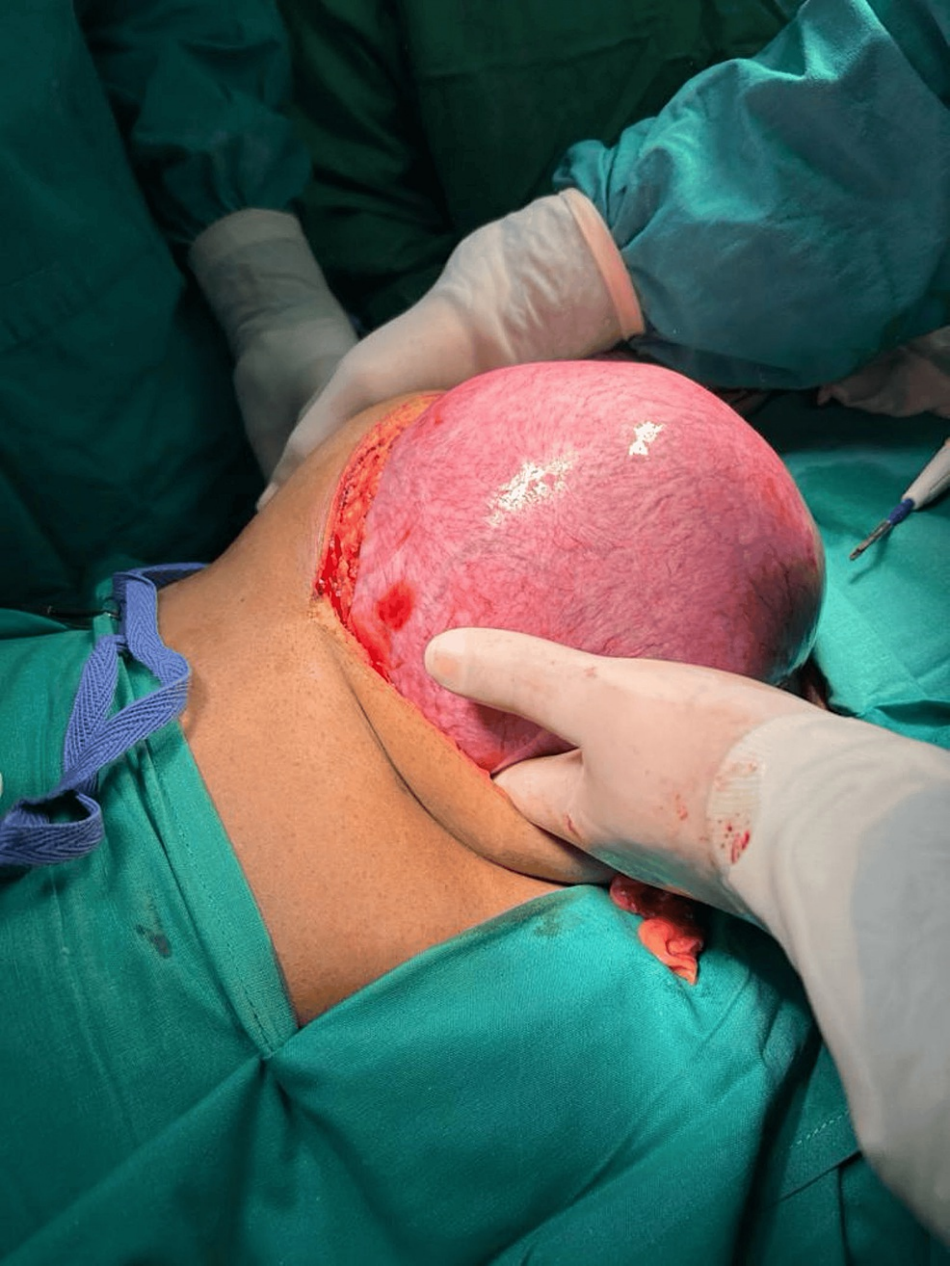
Exploratory laparotomy showing giant uterine leiomyoma The given figure is the original work of authors and has not been sourced from elsewhere.

**Figure 3:**
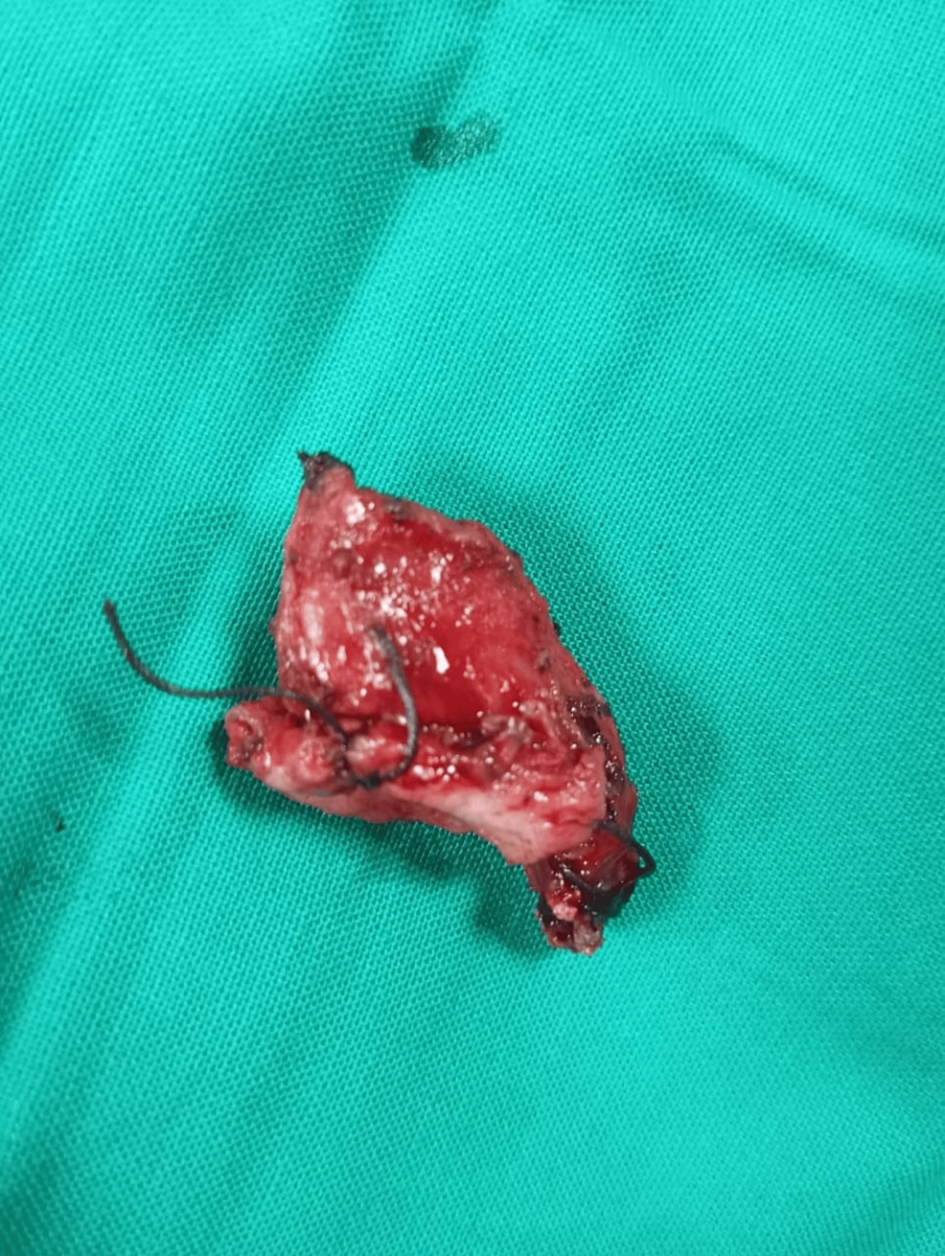
Rudimentary remnant of uterus The given figure is the original work of authors and has not been sourced from elsewhere.

**Figure 4:**
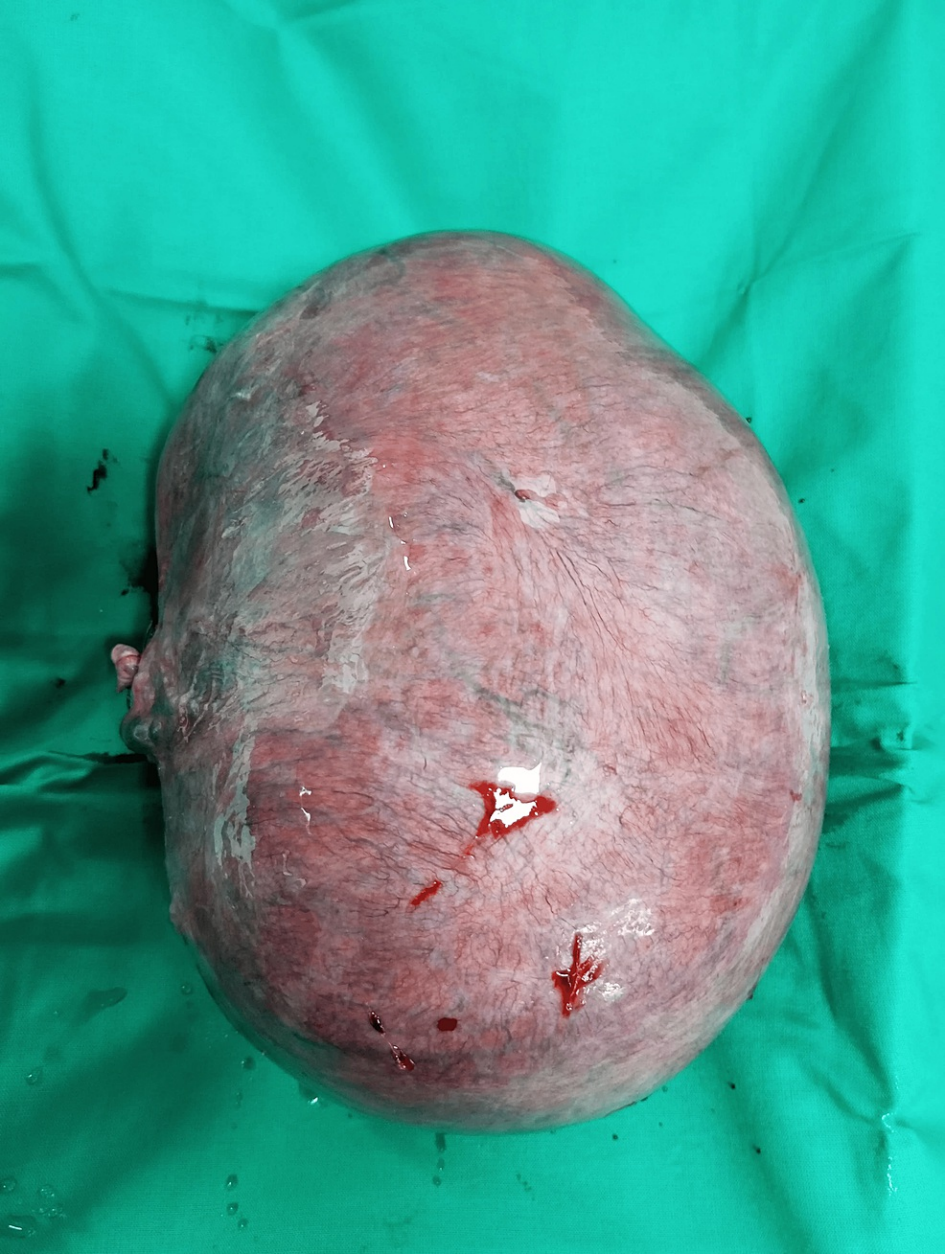
Giant uterine leiomyoma (excised) The given figure is the original work of authors and has not been sourced from elsewhere.

Three forms of CAH exist - salt-wasting, simple virilizing, and nonclassical late-onset disease. The frequency of genital ambiguity is 1:2,000-1:4,500 [15]. This case suggests simple virilizing CAH, characterized by low but detectable 21OH enzyme activity due to a point mutation [16]. Here, we have provided images from the procedure that was performed (Figures 1-4).

Follow up

Patients should be referred to endocrinology for CAH treatment in order to prevent the development of adrenal neoplasia, particularly myelolipoma secondary to increased adrenocorticotropic hormone (ACTH) [3,4]. Prostate specific antigen (PSA) levels for carcinoma prostate [5]. Genetic testing and hormonal profile would also be beneficial. In CAH, rare cases show prostatic tissue in 46, XX females, leading to a male-like phenotype with ambiguous genitalia and precocious puberty. These people tend to have erections and other male secondary sex characteristics, which poses a diagnostic challenge in medical practice [5,6].

## Discussion

In untreated women with CAH, the excess of estrogen results from androgen aromatization [13,17]. Studies showed that in such patients, both aromatase (CYP19A1) and 17β-hydroxysteroid dehydrogenase type I (HSD17B1) were overexpressed in the fibroid tissue compared with the myometrium [10]. CAH can cause masculinization, with genetic factors involving estrogen and progesterone receptors highly expressed [3]. Untreated CAH in XX individuals may experience leiomyoma due to elevated hormones. High androgen levels may stimulate estrogen-dependent organ growth, emphasizing the importance of CAH treatment and screening to prevent complications [4,12,14]. High testosterone with high estradiol was related to an increased risk of giant fibroids in middle-aged women [9,10]. Here we have the Prader scale of virilization, which is used to measure the degree of virilization of external genitalia in individuals with CAH (Table 2) [15-17].

**Table 2:** Prader scale (for degree of virilization)

Prader stage	Degree of virilization
Type 1 (P1)	Clitoral hypertrophy
Type 2 (P2)	Clitoral hypertrophy, urethral and vaginal orifices present, but very close
Type 3 (P3)	Clitoral hypertrophy, single urogenital orifice, posterior fusion of labia majora
Type 4 (P4)	Penile clitoris, perineoscrotal hypospadiasis, complete fusion of labia majora
Type 5 (P5)	Complete masculinization (normal looking male genitalia), but no palpable testes

Treating the largest giant uterine leiomyomas surgically is infrequent and demanding. Past studies have highlighted the dangers of substantial bleeding and perioperative mortality. Although surgery remains the optimal approach, its execution necessitates meticulous planning and intricate perioperative care [9]. Compelling evidence indicates that estrogen plays a pivotal role in the development and proliferation of leiomyomas. In untreated individuals with CAH, estrogen surplus arises from the aromatization of androgens. Studies have revealed heightened expression of aromatase (CYP19A1) and 17β-hydroxysteroid dehydrogenase type I (HSD17B1) in fibroid tissue compared to the myometrium among such patients. This observation implies that leiomyoma cells metabolize circulating androstenedione into estrone (via aromatase), subsequently converting it into the active estrogen, estradiol (via HSD17B1) [2,3].

**Figure 5:**
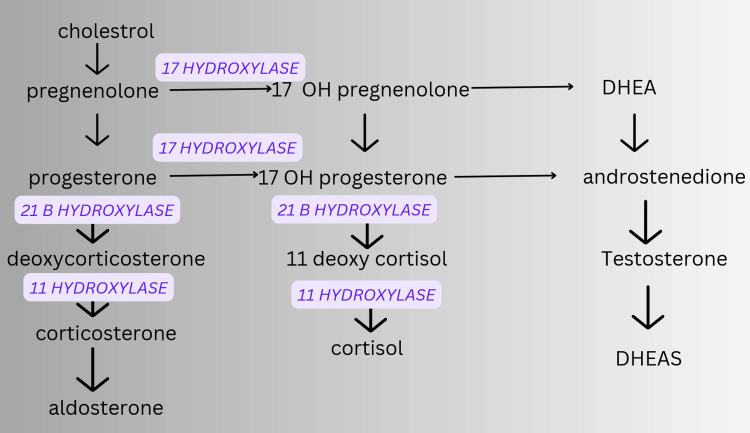
Biochemistry involved in the development CAH CAH; Congenital Adrenal Hyperplasia This figure is the original work of the authors and is not sourced from elsewhere.

Numerous crucial pathways and mechanisms, including those involving sex hormones, extracellular matrix (ECM), Wnt/β-catenin, TGF-β, growth factors, epigenetic and epitranscriptomic regulation, YAP/TAZ, Rho/ROCK, and DNA damage repair pathways, play a role in the development of uterine fibroids [18]. Following is the representation of the mechanisms involved in the biochemistry of CAH (Figure 5) [1].

## Conclusions

In summary, while both uterine leiomyomas and TARTs may occur in individuals with CAH, they involve different organs (uterus vs. testes) and require distinct management strategies. Uterine leiomyomas are treated based on symptoms and reproductive goals, while TARTs require optimization of hormone replacement therapy and, in some cases, surgical intervention.

Sex development relies on the SRY gene for male differentiation (Wolffian ducts) and its absence for females (Mullerian ducts). Disorders encompass atypical genital development categorized into chromosomal, gonadal, and anatomical abnormalities. Anti-Müllerian hormone (AMH) from SRY represses female development. CAH is a highly complex disorder with a myriad of presentations. It is important to have an open approach when encountering a patient with such conditions, as one may approach the case with a different mindset than what is actually the case.
